# Stability and hydrogen storage potential of zirconium-based A_2_ZrH_6_ (A = Na, K) hydrides: a DFT and AIMD investigation

**DOI:** 10.1039/d6ra00660d

**Published:** 2026-03-06

**Authors:** Muhammad Kaleem, Amna Nasir, Zahid Sarfraz, Sanober Kanwal, Asif Nawaz Khan, A. F. Abd El-Rehim, Heba Y. Zahran

**Affiliations:** a Material Research Laboratory (MRL), Department of Physics, International Islamic University H-10 Islamabad 44000 Pakistan mkaleemphy@gmail.com; b Advanced Materials Processing Laboratory, Department of Physics, Air University Pakistan; c Research in Modeling and Simulation Group, COMSATS University Islamabad Pakistan; d Materials Modeling and Simulation Lab, Department of Physics, University of Science & Technology Bannu 28100 Khyber Pakhtunkhwa Pakistan; e Physics Department, Faculty of Science, King Khalid University P. O. Box 9004 Abha 61413 Saudi Arabia

## Abstract

Perovskite-based materials offer considerable potential for efficient, stable and environmentally sustainable hydrogen storage technologies. In this work an inclusive density functional theory (DFT) investigation was conducted to evaluate the structural, mechanical, optoelectronic and thermodynamic features of A_2_ZrH_6_ (A = Na, K) perovskite hydrides. Structural analysis reveals the stable cubic *Fm*3̄*m* symmetry, supported by favorable tolerance factors (0.92–0.99) and negative formation energies endorsing thermodynamic stability. *Ab initio* molecular dynamics (AIMD) simulations assure thermal stability at 300 K without significant structural distortion. Na_2_ZrH_6_ and K_2_ZrH_6_ exhibit notable hydrogen storage characteristics, achieving 4.22 and 3.45 wt% capacities with desorption temperatures of 441.39 and 258.91 K respectively. Mechanical analysis confirms elastic and Born stability with Poisson's ratios of 0.14 (Na_2_ZrH_6_) and 0.25 (K_2_ZrH_6_) and *B*/*G* ratios of 1.06 and 1.66 indicating brittle behavior. Electronic structure calculations confirm the band gaps of 1.25 and 1.87 eV while optical investigations indicate the suitability of these hydrides for photovoltaic applications. These results highlight A_2_ZrH_6_ (A = Na, K) perovskite hydrides as viable and efficient materials for next-generation hydrogen storage and energy conversion systems.

## Abstract 2

Perovskite-based materials offer considerable potential for efficient, stable and environmentally sustainable hydrogen storage technologies.

## Graphical abstract

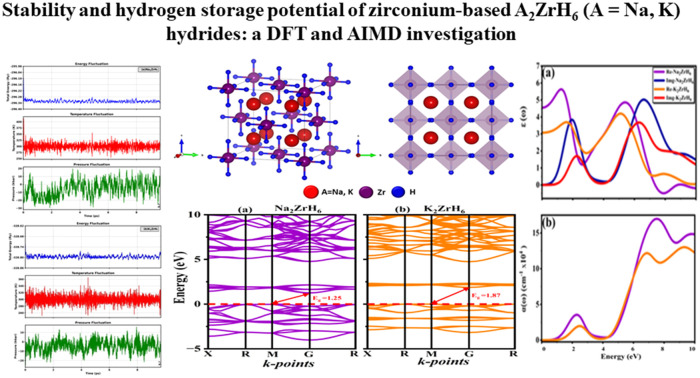

## Introduction

Concerns about the enduring viability of fossil fuel based energy systems have grown as a result of the sharp increase in the world energy demand brought on by industrialization and population expansion.^1–3^ Elongated reliance on coal and oil and natural gas has a negative impact on the environment, contributing to climate change and deteriorating air quality.^4^ These difficulties have sped up the worldwide hunt for sustainable and clean energy sources that can provide future energy needs with little negative influence on the environment.^5^ Due to its high gravimetric energy density, toxic free nature and carbon free combustion products, hydrogen has become a particularly appealing alternative.^6,7^ Despite these benefits, the absence of safe, portable and effective hydrogen storage technology continues to be a major barrier to the widespread use of hydrogen energy. It is commonly acknowledged that resolving this issue is one of the most important obstacles to achieving a hydrogen-based energy economy.^8,9^

Solid-state storage of complex metal hydrides has attracted a lot of attention due to its improved safety, high volumetric density and advantageous thermodynamic features as compared to gaseous and liquid hydrogen storage methods. Because of their highly adjustable physicochemical characteristics and structurally flexible frameworks, perovskite and double perovskite hydrides in this family have attracted increased attention.^10,11^ The perovskite design enables significant compositional engineering through cation substitution which enables systematic control over hydrogen concentration, lattice stability and electrical performance. Specifically organized octahedral networks made of double perovskite hydrides with the general formula A_2_BB′H_6_ are intriguing options for solid state H_2_ storage applications because they can tolerate high H_2_ concentrations without losing structural integrity.^12^

Various double perovskite hydrides have shown aptitude for H_2_ storage in recent theoretical and experimental studies. Research showed that XAlH_6_ (X = Ca, Sr, Ba)^13^ and Na_2_LiXH_6_ (X = Al, Sc, Ga)^2^ and K_2_LiAlH_6_ (ref. 6) demonstrate encouraging H_2_ storage capacities and structural stability. Other related systems such as AeSiH_3_ (Ae = Li, Na, K, Mg),^14^ XSrH_3_ (ref. 15) and A_2_BH_6_ type hydrides have also been examined, revealing diverse electronic, mechanical and thermodynamic behaviors. Recent first-principles studies have further highlighted the potential of double perovskite hydrides for hydrogen storage. For instance Bakar *et al.* investigated double hydrides Na_2_YH_6_ (Y = Ca, Ti) using density functional theory and reported gravimetric H_2_ storage capacities of 6.17 wt% for Na_2_CaH_6_ and 5.72 wt% for Na_2_TiH_6_.^16^ Their electronic structure analysis indicated metallic behavior in Na_2_CaH_6_ whereas Na_2_TiH_6_ exhibited semiconducting character with a band gap of 0.921 eV. Similarly Hakami *et al.* examined the H_2_ storage, mechanical and optoelectronic properties of A_2_FeH_6_ (A = Be, Mg) double perovskite hydrides reporting gravimetric hydrogen capacities of 7.50 wt% for Be_2_FeH_6_ and 5.43 wt% for Mg_2_FeH_6_ along with wide band gaps of 2.89 and 3.08 eV respectively.^17^ In another related study, Ahmed *et al.* explored X_2_FeH_6_ (X = Ca, Sr) hydrides and demonstrated thermodynamic stability and semiconducting behavior with indirect band gaps of 1.67 eV for Ca_2_FeH_6_ and 1.37 eV for Sr_2_FeH_6_ while reporting gravimetric H_2_ densities of 4.28 wt% and 2.54 wt% respectively.^18^

Despite these advances many reported perovskite hydrides continue to face intrinsic limitations including elevated hydrogen desorption temperatures, sluggish sorption kinetics, limited gravimetric capacity or structural instability under operating conditions.^19^ In several instances improvements in H_2_ capacity have been achieved at the expense of mechanical or dynamical stability underscoring the persistent challenge of balancing hydrogen density with material robustness.^20,21^ Motivated by these considerations zirconium based perovskite hydrides represent an attractive yet relatively underexplored class of materials. Zirconium as a tetravalent transition metal provides favorable charge balance and strong metal–hydrogen interactions that can stabilize corner-sharing [ZrH_6_] octahedra within a perovskite lattice. Alkali metal substitution at the A-site offers an additional degree of freedom to tune lattice dimensions, electronic structure and hydrogen binding strength. In particular the A_2_ZrH_6_ (A = Na, K) family is of significant interest as replacing Na^+^ with the larger K^+^ cation is expected to systematically influence structural parameters, thermodynamic stability and hydrogen storage performance. Nevertheless a comprehensive investigation of the structural, mechanical, AIMD stability, H_2_ storage, optoelectronic and thermodynamic properties of Na_2_ZrH_6_ and K_2_ZrH_6_ remains absent from the literature.

In this work a detailed first-principles investigation of A_2_ZrH_6_ (A = Na, K) perovskite hydrides is performed using density functional theory. The study systematically examines their crystal structure, AIMD stability, elastic and mechanical behavior, electronic band structure and density of states, optical, thermodynamic characteristics and H_2_ storage performance to establish clear composition-property relationships. By elucidating how alkali metal substitution impacts lattice stability, electrical responsiveness and hydrogen binding behavior this work identifies crucial factors regulating H_2_ absorption and release in zirconium based perovskite hydrides. The results provide new theoretical insights into this mostly unexplored material class as well as a rational foundation for the design and experimental development of superior solid state H_2_ storage materials. Future studies will also explore the influence of other alkali metals on these material properties, further broadening the scope of perovskite hydride research.

### Computational methodology

The structural and physical characteristics of the A_2_ZrH_6_ (A = Na, K) perovskite hydrides were investigated using first principles calculations carried out within the context of DFT. The CASTEP code which uses a plane wave pseudopotential method to solve the KohnSham equations was used to carry out the simulations. Vanderbilt ultrasoft pseudopotentials were used to represent electron and ion interactions allowing for an accurate and effective handling of valence electrons. The generalized gradient approximation (GGA) was used to tackle the exchange correlation effects in the Perdew–Burke–Ernzerhof (PBE) formulation.^22–24^ The valence electronic configurations explicitly considered in the calculations were Na (2s^2^ 2p^6^ 3s^1^), K (3s^2^ 3p^6^ 4s^1^), Zr (4s^2^ 4p^6^ 4d^2^ 5s^2^) and H (1s^1^). To guarantee consistent convergence of total energies and stress tensors a plane wave kinetic energy cutoff of 600 eV was used. A Monkhorst–Pack *k*-point mesh of 6 × 6 × 6 was used for Brillouin zone integrations and it was confirmed to yield converged structural and electrical properties.^25^ The Broyden–Fletcher–Goldfarb–Shanno (BFGS) minimization approach was used for structural optimizations enabling the simultaneous relaxation of internal atomic coordinates and lattice parameters. The convergence criteria for geometry optimization were set to 2.0 × 10^−5^ eV per atom for total energy 0.05 eV Å^−1^ for maximum force and 2 × 10^−3^ Å for maximum atomic displacement and 0.01 GPa for residual stress.^26^ All calculations were conducted under zero-temperature and zero-pressure conditions to determine the ground-state configurations of the investigated hydrides.

To assess thermal stability beyond static calculations *ab initio* molecular dynamics (AIMD) simulations were performed using the pw.x module of Quantum ESPRESSO. The simulations were conducted within the canonical NVT ensemble with temperature control achieved through a Berendsen thermostat set at 300 K. Atomic trajectories were propagated using the velocity-Verlet algorithm with a time step of 0.97 fs over 10 000 steps corresponding to a total simulation time of approximately 9.7 ps. Ultrasoft pseudopotentials consistent with the PBE exchange-correlation functional were employed with plane-wave and charge-density cutoffs of 60 Ry and 400 Ry respectively. The self-consistent field convergence threshold was set to 1.0 × 10^−8^ Ry while total energy and force convergence criteria were fixed at 1.0 × 10^−5^ Ry and 1.0 × 10^−4^ Ry/Bohr respectively.^27^

## Results and discussion

### Structural properties

**Fig. 1:**
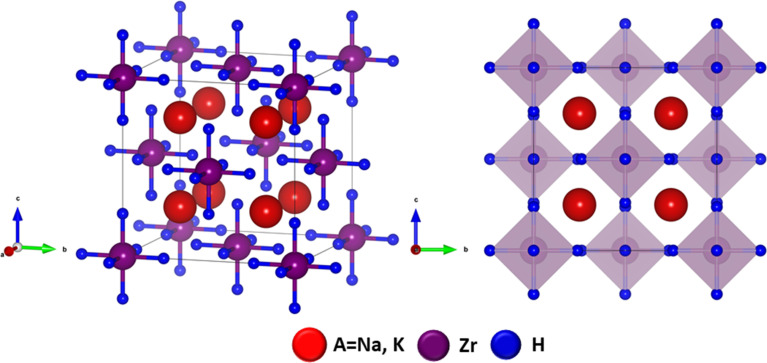
Optimized crystal structure of A_2_ZrH_6_ (A = Na, K) perovskite hydrides.

The structural characteristics and geometric stability of the A_2_ZrH_6_ (A = Na, K) perovskite hydrides were systematically investigated using first-principles DFT calculations. Both compounds crystallize in a highly symmetric cubic structure with space group *Fm*3̄*m* (No. 225) preserving the ideal perovskite framework.^28^ The atomic arrangements within the cubic unit cell follow distinct Wyckoff positions in which the A-site cations (Na or K) occupy the 8c sites (0.25, 0.25, 0.25) while Zr atoms are positioned at the 4a sites (0, 0, 0) forming the backbone of the perovskite lattice. The hydrogen atoms be vested at the 24e sites (*x*, 0, 0) where the internal parameter *x* = 0.245 resulting in marginally distorted ZrH_6_ octahedra as displayed in Fig. 1. This octahedral coordination directly affects hydrogen binding and is essential for maintaining the crystal structure. The optimized lattice constants were found to be 8.31 Å for Na_2_ZrH_6_ and 8.95 Å for K_2_ZrH_6_ with lattice parameters satisfying *a* = *b* = *c* as summarized in Table 1. The observed lattice expansion upon substitution of Na with the larger K cation is further reflected in the computed unit cell volumes which increase from 573.87 Å^3^ for Na_2_ZrH_6_ to 718.86 Å^3^ for K_2_ZrH_6_ consistent with the ionic size difference between Na^+^ and K^+^.

**Table 1:** Structural parameters of A_2_ZrH_6_ (A = Na, K) perovskite hydrides

Parameters	Na_2_ZrH_6_	K_2_ZrH_6_	Sr_2_FeH_6_ (ref. 18)	Ca_2_FeH_6_ (ref. 18)	Sr_2_VH_6_ (ref. 30)
Lattice constant *a* = *b* = *c* (Å)	8.31	8.95	5.30	4.87	7.12
Volume (Å)^3^	573.87	718.86	148.88	115.50	59.56
*τ*_G_	0.92	0.99	—	—	0.99
*µ*	0.47	0.47	—	—	0.47
Δ*H*_*f*_	−1.33	−0.78	−4.23	−6.93	−0.284
Δ*H*_f_(kJ mol^−1^ H_2_)	−57.69	−33.84	—	—	−33.84
C_wt%_	4.22	3.45	2.54	4.28	2.60
*T*_des_ (K)	441.39	258.91	—	—	210.7

To further assess geometric stability, the Goldschmidt tolerance factor (*τ*_G_) and octahedral factor (*µ*) were evaluated using the eqn (1) and (2):^29^1
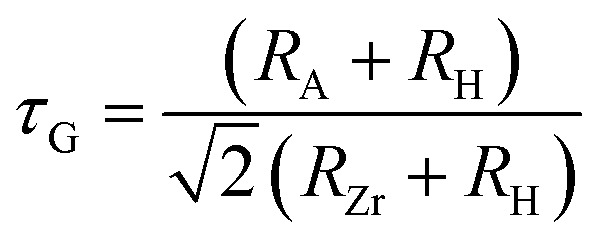
2
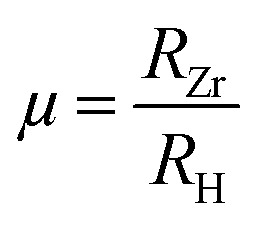
where *R*_A_, *R*_Zr_ and *R*_H_ denote the ionic radii of the A-site cation (Na or K), Zr and hydrogen respectively. As listed in Table 1 the calculated *τ*_G_ values are 0.92 for Na_2_ZrH_6_ and 0.99 for K_2_ZrH_6_ both lying within the accepted stability range for cubic perovskites. The octahedral factor *µ* was found to be 0.47 for both compounds which falls well within the conventional stability window confirming the robustness of the ZrH_6_ octahedral network.

Moreover the thermodynamic stability of the investigated compounds was further examined through the calculation of the formation energy (Δ*H*_f_) given by eqn (3):^31^3
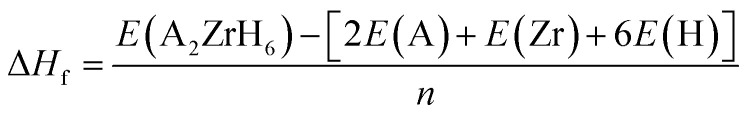
where *E*(A_2_ZrH_6_) is the energy of the compound *E*(A), *E*(Zr) and *E*(H) represent the total energies of the isolated constituent atoms and *n* is the total number of atoms in the formula unit. The calculated formation energies are −1.33 eV per atom (−57.69 kJ mol^−1^ H_2_) for Na_2_ZrH_6_ and −0.78 eV per atom (−33.84 kJ mol^−1^ H_2_) for K_2_ZrH_6_ confirming the exothermic formation and intrinsic thermodynamic stability of both perovskite hydrides. These structural features combined with favorable geometric and thermodynamic stability imply that A_2_ZrH_6_ (A = Na, K) perovskite hydrides provide a robust framework for reversible H_2_ absorption and release supporting its potential application in solid state H_2_ storage systems.

### Hydrogen storage

Hydrogen is largely considered as a vital energy vector for achieving a sustainable and low carbon energy future owing to its high gravimetric energy density and environmentally benign utilization. However a significant obstacle to its widespread use is still the absence of safe and portable and effective H_2_ storage systems. In this sense solid state H_2_ storage based on complex metal hydrides has garnered a lot of interest since it provides better safety and volumetric efficiency when compared to traditional gaseous and liquid storage techniques. Perovskite derived hydrides particularly A_2_ZrH_6_ (A = Na, K) systems represent a promising class of materials due to their structural robustness and intrinsically high hydrogen content. The gravimetric hydrogen capacity which measures the mass fraction of hydrogen in relation to the total mass of the storage medium is a crucial metric for assessing the efficacy of hydrogen storage. The eqn (4) was used to get the theoretical gravimetric hydrogen capacity:^32^4
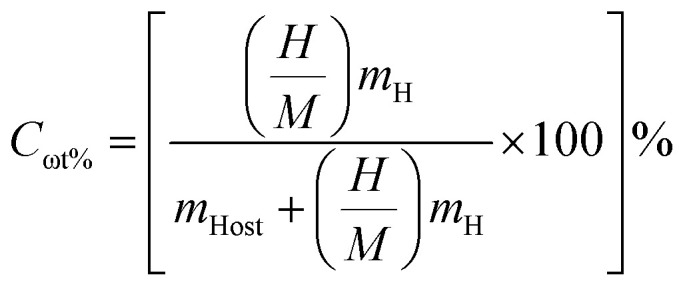
where *m*_Host_ and *m*_H_ represent the molar masses of the host lattice and hydrogen respectively and 
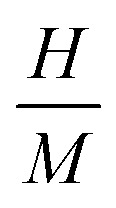
 denotes the hydrogen to metal ratio. Using this formulation the gravimetric hydrogen capacities of Na_2_ZrH_6_ and K_2_ZrH_6_ were determined to be 4.22 wt% and 3.45 wt% respectively as listed in Table 1. The higher capacity of Na_2_ZrH_6_ originates from the lower atomic mass of sodium which enhances the hydrogen-to-host mass ratio. These values are competitive among complex hydride systems and show a clear possibility for more optimization through compositional adjustment even if they are still below the U.S. DOE 2025 gravimetric target of 5.5 wt%. In addition to storage capacity the hydrogen release behaviour plays a crucial role in practical applications and is commonly assessed through the desorption temperature (*T*_des_). Desorption temperature can be estimated from thermodynamic considerations based on the Gibbs free energy eqn (5): ^33,34^5
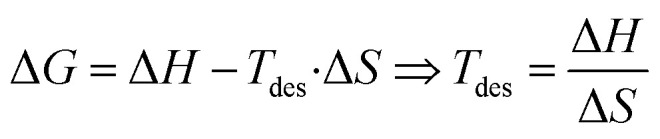
where Δ*H* and Δ*S* denote the enthalpy and entropy changes associated with hydrogen desorption. The computed desorption temperatures for Na_2_ZrH_6_ and K_2_ZrH_6_ using the standard entropy of H_2_ gas are 441.39 K and 258.91 K respectively (Table 1). While the greater *T*_des_ of Na_2_ZrH_6_ indicates stronger metal hydrogen contacts and improved thermal stability the somewhat lower desorption temperature of K_2_ZrH_6_ indicates more advantageous hydrogen release under moderate operating conditions. It is important to note that these values are rough estimates based on the standard entropy of gaseous hydrogen. Further experimental and theoretical studies are needed to refine these estimates and provide a more accurate understanding of the hydrogen desorption process. When combined, these findings show that in A_2_ZrH_6_ (A = Na, K) perovskite hydrides gravimetric capacity and H_2_ release properties are balanced. While K_2_ZrH_6_ shows more accessible desorption behavior Na_2_ZrH_6_ gives a larger H_2_ storage capacity indicating the potential for compositional engineering to attain optimal H_2_ storage performance. In comparison to other hydrogen storage materials like MgH_2_,^35^ which has a gravimetric hydrogen capacity of 7.6 wt%, Na_2_ZrH_6_ and K_2_ZrH_6_ show lower capacities. However, Na_2_ZrH_6_ offers a better balance between hydrogen storage capacity and thermal stability, with a desorption temperature (*T*_des_) of 441.39 K, which is higher than K_2_ZrH_6_ (258.91 K), making it more stable under high-temperature conditions. K_2_ZrH_6_, however, exhibits more accessible hydrogen desorption behavior at lower temperatures, making it more favorable for use under moderate operating conditions.

### *Ab Initio* molecular dynamics (AIMD) calculations

**Fig. 2:**
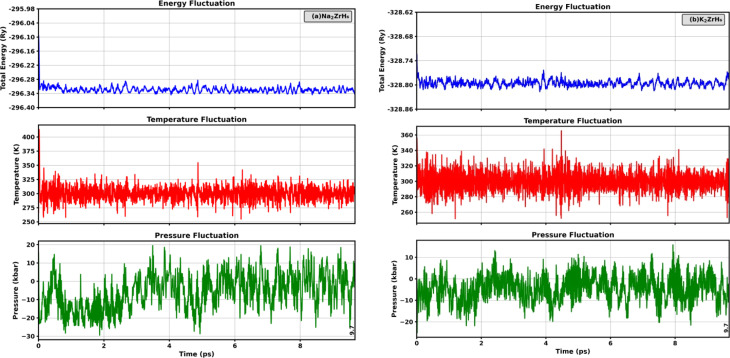
AIMD total-energy traces *versus* time for (a) Na_2_ZrH_6_ and (b) K_2_ZrH_6_ hydrides.

AIMD simulations conducted at room temperature were used to investigate the dynamical and thermal stability of the A_2_ZrH_6_ (A = Na, K) perovskite hydrides as illustrated in Fig. 2(a and b). AIMD simulations provide a realistic assessment of finite temperature behavior by merging first principles electronic structure computations with classical atomic motion and directly evaluating total energy development and thermodynamic stability under thermal agitation.^36^ The total energy profile for Na_2_ZrH_6_ is well controlled over the 9.7 ps simulation frame with extremely small amplitude fluctuations around an equilibrium value as seen in Fig. 2(a). Importantly no systematic drift or sudden fluctuation is seen indicating that there is no structural instability during heat stimulation. The temperature evolution continuously varies about the target value of 300 K while remaining within a narrow physically attainable range. This behavior shows that under ambient conditions the Na_2_ZrH_6_ framework does not undergo phase transformation or breakdown and retains its structural integrity. As seen in Fig. 2(b) K_2_ZrH_6_ reacts similarly. A dynamically stable lattice is suggested by the total energy small fluctuations and lack of abrupt discontinuities throughout the trial. With brief fluctuations typical of constant temperature AIMD simulations the associated temperature swings stay centered around room temperature. The mechanical system of K_2_ZrH_6_ and thermal endurance are further supported by the lack of abnormal pressure or temperature variations. These findings which consistently show limited energy routes and steady temperature profiles offer compelling evidence of the thermal and dynamical stability of both Na_2_ZrH_6_ and K_2_ZrH_6_. These perovskite hydrides resilience under practical operating settings is demonstrated by the AIMD simulations lack of structural degradation which further supports their applicability for H_2_ storage and energy related applications.

### Mechanical properties

Mechanical characteristics exhibit a decisive role in prevailing the elastic response and structural reliability of perovskite hydrides mainly under the cyclic stresses associated with H_2_ absorption and release.^37^ A quantitative evaluation of elastic constants is therefore essential to assess mechanical stability and deformation behavior.^38^ In this work the second-order elastic constants (*C*_*ij*_) of A_2_ZrH_6_ (A = Na, K) perovskite hydrides were calculated from which key mechanical parameters were derived. The calculated elastic constants and related mechanical properties are summarized in Table 2. Due to the cubic crystal symmetry only three independent elastic constants *C*_11_, *C*_12_ and *C*_44_ are required to describe the elastic behavior of the studied compounds.^39^ These constants represent resistance to uniaxial deformation, transverse deformation and shear deformation respectively. The calculated values for both Na_2_ZrH_6_ and K_2_ZrH_6_ satisfy the Born-Huang mechanical stability criteria for cubic crystals: *C*_11_ − *C*_12_ > 0, *C*_11_ + 2*C*_12_ > 0, *C*_11_ > 0 and *C*_44_ > 0 confirming that both compounds are mechanically stable under small strains. As shown in Table 2 Na_2_ZrH_6_ exhibits relatively lower elastic stiffness with *C*_11_ = 19.48 GPa, *C*_12_ = 4.41 GPa and *C*_44_ = 9.96 GPa whereas K_2_ZrH_6_ displays higher longitudinal rigidity with *C*_11_ = 54.75 GPa indicating stronger resistance to uniaxial deformation. The larger *C*_11_ value of K_2_ZrH_6_ reflects enhanced stiffness along principal crystallographic directions consistent with its expanded lattice framework.

**Table 2:** Elastic constants calculated for A_2_ZrH_6_ (A = Na, K) perovskite hydrides

Elastic moduli	Na_2_ZrH_6_	K_2_ZrH_6_	Sr_2_FeH_6_ (ref. 18)	Ca_2_FeH_6_ (ref. 18)	Sr_2_VH_6_ (ref. 30)
*C*_11_	19.48	54.75	95.38	117.19	67.75
*C*_12_	4.41	6.69	17.32	26.99	12.32
*C*_44_	9.96	9.11	30.14	38.78	21.56
Born's stability	Stable	Stable	Stable	Stable	Stable
*B* (GPa)	9.43	22.71	43.34	57.06	30.79
*G* (GPa)	8.90	13.60	33.42	41.19	23.84
*E* (GPa)	20.32	34.02	79.75	99.60	56.86
*B*/*G*	1.06	1.66	1.29	1.38	1.23
*ν*	0.14	0.25	—	—	0.19
*A* ^U^	0.09	1.22	0.77	0.86	0.778
*C*_p_	−5.55	−2.42	—	—	−9.24

The bulk modulus (*B*) and shear modulus (*G*) were calculated using the Voigt–Reuss–Hill (VRH) approximation in order to assess macroscopic elastic behavior.^40^ The shear modulus shows resistance to shape deformation whereas the bulk modulus shows resistance to homogeneous compression. The calculated *B* values are 9.43 GPa for Na_2_ZrH_6_ and 22.71 GPa for K_2_ZrH_6_ indicating that K_2_ZrH_6_ possesses a higher resistance to volume compression. In a similar vein the shear modulus rises from 8.90 GPa for Na_2_ZrH_6_ to 13.60 GPa for K_2_ZrH_6_ indicating that the K based combination is more stiff. The Young's modulus (*E*) which measures stiffness under uniaxial stress and has values of 20.32 GPa for Na_2_ZrH_6_ and 34.02 GPa for K_2_ZrH_6_ further supports this tendency. These findings show that Na_2_ZrH_6_ is relatively more compliant than K_2_ZrH_6_ which may be helpful for adapting to volume variations during H_2_ absorption and desorption. However, the comparatively low elastic moduli, particularly for Na_2_ZrH_6_, also indicate mechanical softness, meaning that the material can deform more readily under external or internally generated cycling stresses.

Additionally the ductile or brittle nature of the materials was assessed using Pugh ratio (*B*/*G*) and Poisson's ratio (*ν*). According to criteria materials with *B*/*G* > 1.75 and *ν* > 0.26 exhibit ductile nature.^41^ The computed *B*/*G* ratios of 1.06 (Na_2_ZrH_6_) and 1.66 (K_2_ZrH_6_) along with Poisson's ratios of 0.14 and 0.25 respectively indicate that both compounds exhibit a predominantly brittle mechanical character with K_2_ZrH_6_ lying close to the brittle or ductile transition. Elastic anisotropy was quantified using the universal anisotropy index (*A*^U^). The low value of *A*^U^ = 0.09 for Na_2_ZrH_6_ suggests near isotropic elastic behavior whereas the higher value of A^U^ = 1.22 for K_2_ZrH_6_ indicates pronounced elastic anisotropy and direction dependent deformation characteristics. Additional insight into bonding nature was obtained from the Cauchy pressure (*C*_p_ = *C*_12_ − *C*_44_) which yields negative values of −5.55 GPa for Na_2_ZrH_6_ and −2.42 GPa for K_2_ZrH_6_. The unfavorable brittle mechanical behavior in hydride systems is frequently linked to a directed and covalent bonding character, as suggested by Cauchy pressures. From a practical perspective, the combination of low stiffness and brittleness presents both advantages and challenges for hydrogen cycling. While the low moduli can help absorb volumetric strain during repeated H_2_ absorption and desorption, reducing stress buildup, the brittle nature may lead to crack formation, particle fragmentation, and loss of structural integrity over time, especially under uneven stresses or microstructural defects. Thus, although the current elastic analysis confirms stability under small strains, a full assessment of cycling durability would require further studies focused on fracture behavior and microstructural impacts, which are beyond the scope of this work. Overall, the calculated elastic parameters indicate that A_2_ZrH_6_ (A = Na, K) perovskite hydrides are mechanically stable under small strains, exhibit moderate stiffness, and predominantly brittle behavior, which should be considered when interpreting their ability to withstand structural stresses during repeated hydrogen absorption and release cycles, as shown in the elastic constants and derived mechanical parameters in Table 2.

### Electronic properties

**Fig. 3:**
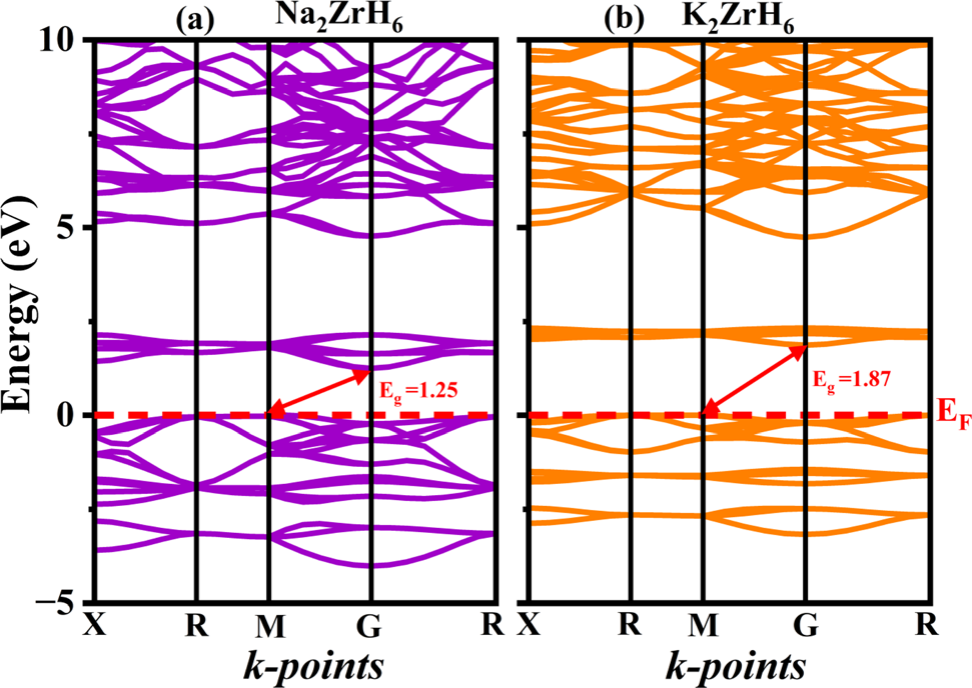
Computed band structures of (a) Na_2_ZrH_6_ and (b) K_2_ZrH_6_ perovskite hydrides.

Electronic characteristics play a central role in governing hydrogen adsorption–desorption kinetics, charge redistribution and chemical stability in complex hydrides. In perovskite hydrides the magnitude and nature of the electronic band gap together with the distribution of electronic states near the Fermi level directly influence hydrogen binding strength and diffusion pathways.^42,43^ To elucidate these effects the electronic band structures and DOS of A_2_ZrH_6_ (A = Na, K) were systematically analyzed using first principles calculations. The calculated electronic band structures of Na_2_ZrH_6_ and K_2_ZrH_6_ along the high symmetry directions X–R–M–Γ–R are presented in Fig. 3(a and b) with the Fermi level (*E*_F_) aligned at 0 eV. A substantial separation between the valence and conduction bands characterizes these materials semiconducting nature. Na_2_ZrH_6_ has an indirect band gap of about 1.25 eV because the valence band maximum and conduction band minimum occur at distinct symmetry sites. This restrained band gap validates that there is enough electronic insulation to prevent undesired electronic leakage while permitting charge redistribution during H_2_ absorption and release. K_2_ZrH_6_ on the other hand exhibits a broader indirect band gap of roughly 1.87 eV which is a result of the greater K^+^ ionic radius expanding the lattice and decreasing orbital overlap inside the Zr–H framework. During repeated hydrogen cycling, the increasing band gap suggests improved electronic stability which may be advantageous for preserving structural integrity.^44^ The fact that both hydrides are semiconducting implies that electron transport is regulated rather than metallic. Improved reversibility and fewer parasitic processes in H_2_ storage materials are often associated with this feature.

**Fig. 4:**
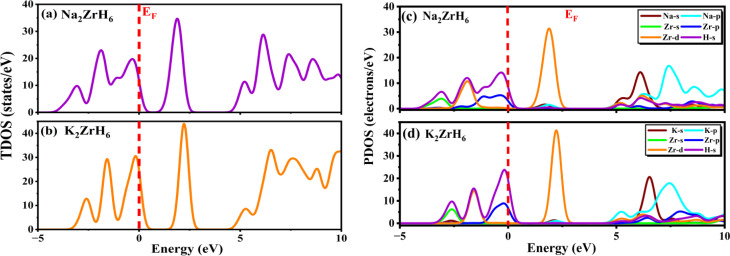
(a–d): TDOS and PDOS of A_2_ZrH_6_ (A = Na, K) perovskite hydrides.

The total density of states (TDOS) displayed in Fig. 4(a and b) provides further information on the electrical response. The TDOS shows a significant depletion of states at the Fermi level in both Na_2_ZrH_6_ and K_2_ZrH_6_ supporting the band structure findings and verifying their semiconducting nature. The lack of finite DOS at *E*_F_ suggests electronic passivation which reduces electronic contributions to structural degradation under operating conditions and aids in stabilizing the H_2_ rich lattice. The TDOS peaks pronounced asymmetry near the Fermi level indicates that the effective masses of electrons and holes are different. Reaction kinetics may be impacted by this asymmetry potential to affect charge redistribution during hydrogen uptake and release.

The partial density of states (PDOS) for Na_2_ZrH_6_ and K_2_ZrH_6_ displayed in Fig. 4(c and d) reveals the orbital origins of the electronic bands. In both compounds the valence band region below *E*_F_ is dominated by H-s states with appreciable hybridization from Zr-d and alkali metal s/p orbitals. This hybridization reflects mixed ionic-covalent bonding within the ZrH_6_ octahedra which is essential for reversible hydrogen binding. Above the Fermi level the conduction bands are primarily governed by Zr-4d states with secondary contributions from Na-3p or K-4p orbitals. The stronger Zr-d character in the conduction manifold indicates that electronic excitation and charge transfer during hydrogen release are mainly mediated through the Zr–H network. Compared to Na_2_ZrH_6_, K_2_ZrH_6_ shows a slightly reduced overlap between Zr-d and H-s states near the band edges consistent with its larger band gap and expanded lattice.

Both Na_2_ZrH_6_ and K_2_ZrH_6_ are indirect gap semiconductors with band gaps falling in a region that combines electrical stability and bonding flexibility according to the combined band structure and DOS investigations. The ZrH_6_ octahedra play a crucial role in regulating H_2_ retention and release as evidenced by the preponderance of H(s) states in the valence band and Zr(d) states in the conduction band. While retaining enough electronic flexibility to enable reversible sorption processes these electronic characteristics promote stable H_2_ accommodation.

### Optical properties

The optical response provides direct insight into photon matter interaction in semiconducting hydride perovskites and is closely linked to interband transitions across the band gap. In H_2_ storage hydrides such photoexcited carrier generation and associated dielectric screening might promote charge redistribution and localized heating supporting surface reaction steps and influencing absorption desorption rates.^45^ This connection between optical properties and hydrogen storage/release performance is important, as the photo-induced carrier excitation can enhance charge redistribution, which supports the surface reaction steps crucial for hydrogen release.

**Fig. 5:**
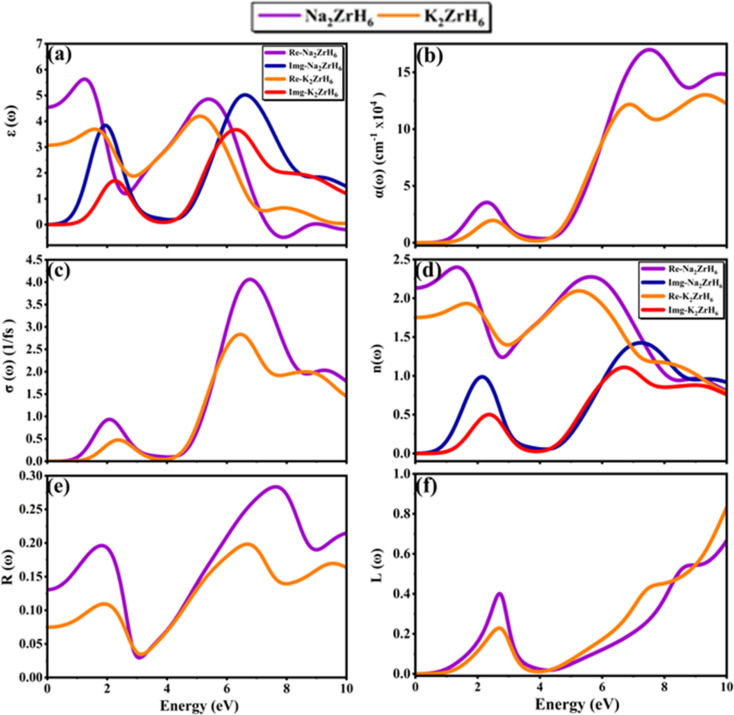
(a–f): The computed graphs of optical parameters (a) real and imaginary *ε*(*ω*) (b) absorption spectrum *α*(*ω*) (c) optical conductivity *σ*(*ω*) (d) refractive index *n*(*ω*) and extinction coefficient *k*(*ω*) (e) reflectivity *R*(*ω*) and (f) loss function *L*(*ω*) for A_2_ZrH_6_ (A = Na, K) perovskite hydrides.

To clarify these effects the frequency dependent optical properties of A_2_ZrH_6_ (A = Na, K) were evaluated. The computed dielectric function and the derived optical spectra are presented in Fig. 5(a–f). The optical response is designated by the complex dielectric function *ε*(*ω*) = *ε*_1_(*ω*) + *ε*_2_(*ω*) where *ε*_1_(*ω*) reflects dispersion (polarization) and *ε*_2_(*ω*) represents absorption due to interband transitions.^46^ As shown in Fig. 5(a) both compounds exhibit finite static dielectric constants indicating measurable low-energy electronic polarizability. Na_2_ZrH_6_ displays a higher static dielectric constant of approximately *ε*_1_(0) 4.6 whereas K_2_ZrH_6_ shows a lower value of about *ε*_1_(0) 3.3 reflecting stronger dielectric screening and enhanced polarization in the Na-based hydride. With increasing photon energy *ε*_1_(*ω*) decreases gradually and exhibits pronounced dispersion features before crossing zero in the 3.5–4.0 eV range for Na_2_ZrH_6_ and slightly above 4.2 eV for K_2_ZrH_6_ indicating the effective plasma frequency and the transition from reflective to transparent behavior. At higher energies *ε*_1_(*ω*) attains weakly negative values followed by stabilization, a response characteristic of semiconductors governed by interband transitions rather than free-carrier (Drude) contributions.

The imaginary part *ε*_2_(*ω*) also shown in Fig. 5(a) exhibits an earlier onset for Na_2_ZrH_6_ at 1.3 eV consistent with its smaller electronic band gap (*E*_g_ = 1.25 eV) while K_2_ZrH_6_ shows a delayed onset near 1.9 eV in agreement with its wider gap (*E*_g_ = 1.87 eV). For Na_2_ZrH_6_*ε*_2_(*ω*) displays prominent absorption peaks centered around 2.6 eV and 5.8 eV with a maximum intensity approaching *ε*_2_ of 5.0 whereas K_2_ZrH_6_ exhibits comparatively weaker peaks near 3.0 eV and 6.2 eV reaching a maximum of *ε*_2_ 4.0. These dominant spectral features originate from interband transitions between H-derived valence states and Zr-d dominated conduction states as corroborated by the DOS analysis.^47^ The stronger *ε*_2_(*ω*) response of Na_2_ZrH_6_ indicates higher transition probability and enhanced photon-induced carrier excitation which can facilitate charge redistribution and support hydrogen desorption processes under optical or thermal stimulation. This enhanced carrier excitation directly influences hydrogen release, with Na_2_ZrH_6_ showing a stronger capability to drive the desorption process through photon stimulation. The absorption coefficient is obtained from the extinction coefficient *k*(*ω*) through equation 
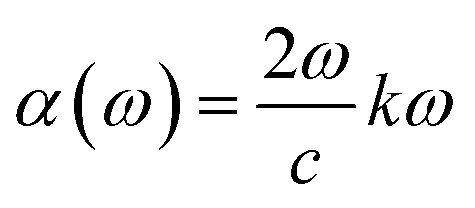
.^48^ In Fig. 5(b) both materials show negligible absorption at very low energies followed by a clear rise after the band-edge region. Na_2_ZrH_6_ exhibits the stronger absorption across the spectrum, including a low-energy feature around 2–3 eV (peak *α* ∼ 3.0 × 10^4^ cm^−1^) and a dominant high-energy band in the near-UV where the absorption reaches *α* ≈ 1.7 × 10^5^ cm^−1^ (around 7–8 eV). In parallel, K_2_ZrH_6_ peaks at *α* ≈ 1.2–1.3 × 10^5^ cm^−1^ in the same region. The stronger *α*(*ω*) for Na_2_ZrH_6_ indicates more intense photon–electron coupling which can support photo-assisted carrier excitation and localized thermal effects relevant to hydrogen release. This stronger coupling can directly facilitate more efficient photon-driven hydrogen desorption.

Optical conductivity *σ*(*ω*) describes the frequency-dependent response of charge carriers under an external electromagnetic field and directly reflects photon-induced electronic transitions that govern photo assisted charge transport and hydrogen desorption dynamics. The optical conductivity can be expressed as 
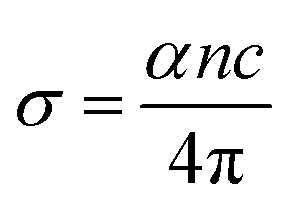
.^49^ In Fig. 5(c) both compounds show conductivity features that track the absorption bands. Na_2_ZrH_6_ reaches a higher peak optical conductivity (4.0 in the plotted units) near 6–7 eV whereas K_2_ZrH_6_ peaks at a lower magnitude (2.8) in the same region confirming stronger photoinduced carrier activity in the Na-based hydride.

The refractive index and extinction coefficient provide further insight into light propagation and attenuation inside the material and are particularly relevant for assessing light-matter interaction strength in semiconducting hydrides. As evident from Fig. 5(d) both Na_2_ZrH_6_ and K_2_ZrH_6_ exhibit relatively high refractive indices in the low-energy region, consistent with their semiconducting nature and finite band gaps. Na_2_ZrH_6_ shows a higher static refractive index, with *n*(0) ≈ 2.1–2.3 compared to *n*(0) 1.7–1.9 for K_2_ZrH_6_ indicating stronger electronic polarization and enhanced photon coupling at low photon energies. The refractive index *n*(*ω*) and extinction coefficient *k*(*ω*) are consequent from the real and imaginary parts of the dielectric function using eqn (6).^50^6
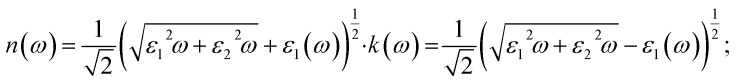


As shown in Fig. 5(d) the refractive index of both compounds gradually decreases with increasing photon energy and approaches unity beyond 7 eV reflecting reduced light-matter interaction in the high energy ultraviolet region. The consistently larger *n*(*ω*) values for Na_2_ZrH_6_ across the visible range indicate stronger dispersion and improved optical confinement which can assist photon-assisted charge excitation relevant for hydrogen release. Significant absorption features in the visible range are revealed by the extinction coefficient spectra which are also shown in Fig. 5(d). While K_2_ZrH_6_ displays a relatively weaker peak with *k*_max_ 0.5 and Na_2_ZrH_6_ displays a prominent extinction peak about 2–3 eV with *k*_max_ 1.0. Both materials exhibit wider extinction bands that extend into the UV region at higher energies which are linked to interband electronic transitions involving Zr-d and H-s states. Stronger electromagnetic wave attenuation and improved absorption efficiency which might encourage localized heating and speed up hydrogen desorption processes are confirmed by the greater extinction coefficient of Na_2_ZrH_6_.

Reflectivity *R*(*ω*) quantifies the fraction of incident electromagnetic radiation reflected from the material surface and provides insight into surface optical response absorption efficiency and photon matter coupling which are relevant for light assisted hydrogen absorption and desorption processes can be calculated using eqn (7).^51^7
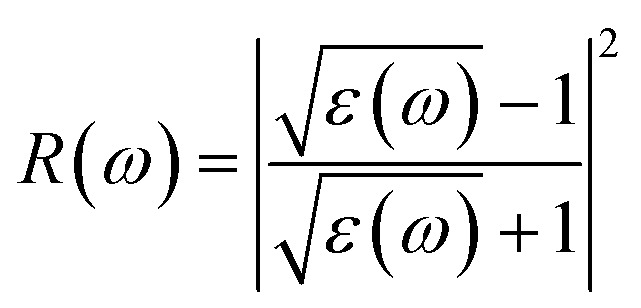


As shown in Fig. 5(e) both materials exhibit low-to-moderate reflectance with a pronounced minimum around 3 eV (down to 0.03–0.05) coincident with strong absorption. At higher energies Na_2_ZrH_6_ attains a larger reflectivity maximum of 0.28 near 7–8 eV while K_2_ZrH_6_ peaks at 0.20 in a similar range indicating a comparatively stronger reflective response of the Na compound in the near-UV. The energy-loss function provides insight into collective electronic excitations and plasmonic behavior describing the energy dissipated by fast electrons traversing the material and serving as a sensitive indicator of dielectric screening and electronic stability and calculated by eqn (8).^52^8
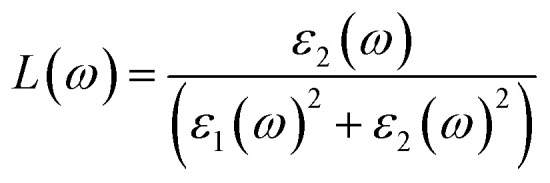


In Fig. 5(f) both compounds show a clear low-energy loss peak near ≈2–3 eV (Na_2_ZrH_6_: *L*_max_ 0.40 and K_2_ZrH_6_: *L*_max_ 0.25) followed by a gradual increase at higher energies. Toward 10 eV K_2_ZrH_6_ rises more strongly (approaching 0.85) than Na_2_ZrH_6_ (0.65) suggesting comparatively higher high energy loss in the K-based hydride.

Overall both A_2_ZrH_6_ (A = Na, K) compounds exhibit semiconducting optical responses with absorption onsets governed by their band gaps and dominant interband activity in the visible-to-UV range. Across Fig. 5(a–f) Na_2_ZrH_6_ consistently shows stronger dielectric screening, higher absorption and larger optical conductivity indicating more efficient photon-driven electronic excitation which can be beneficial for photo-assisted processes and thermally supported hydrogen cycling.

### Thermodynamic properties

Thermodynamic properties play a central role in determining the feasibility and kinetic performance of hydrogen storage materials. Parameters such as sound velocities, Debye temperature (*θ*_D_) and melting temperature (*T*_m_) provide indirect yet reliable insight into lattice stiffness, bond strength, phonon transport and thermal robustness. In H_2_ storage systems these features directly influence hydrogen diffusion kinetics and absorption desorption reversibility and resistance to structural degradation during repeated thermal cycling. In this context elastic constants serve as a fundamental basis for evaluating the thermodynamic stability of A_2_ZrH_6_ (A = Na, K) perovskite hydrides. The longitudinal (*v*_l_) and transverse (*v*_t_) sound velocities were derived from the bulk modulus (*B*) or shear modulus (*G*) and were used to calculate the average sound velocity (*v*_m_). The Debye temperature and melting temperature were subsequently estimated using definite eqn (9)–(11):^53,54^9
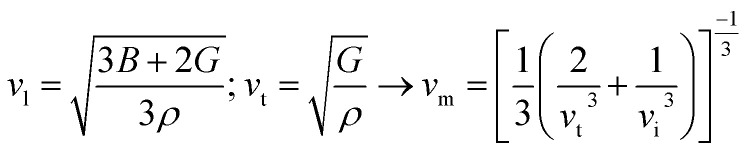
10
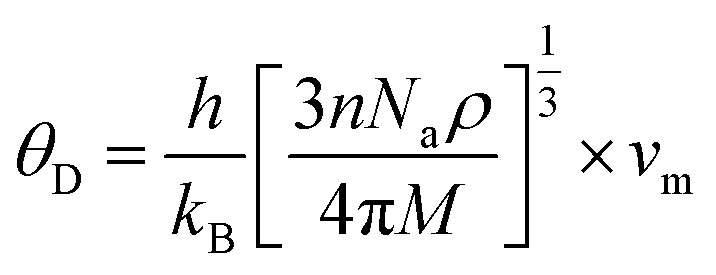

11 *T*_m_ = [553 + 5.911(*C*_11_)] ± 300

where *h* is Planck's constant, *k*_B_ represents Boltzmann's constant and *n* is the number of atoms per formula unit, *N*_a_ is Avogadro's number, *M* is the molecular mass and *C*_11_ is the elastic constant. The calculated thermodynamic parameters for Na_2_ZrH_6_ and K_2_ZrH_6_ are summarized in Table 3. For Na_2_ZrH_6_ the longitudinal and transverse sound velocities are 3.04 km s^−1^ and 2.32 km s^−1^ respectively resulting in an average sound velocity of 2.48 km s^−1^. These values result in a Debye temperature of 293 K which indicates phonon activity and moderate lattice stiffness. Under moderate temperature circumstances the corresponding melting temperature of 668 K indicates adequate thermal stability for H_2_ storage operations. In parallel K_2_ZrH_6_ exhibits enhanced elastic wave propagation with *v*_l_ = 4.43 km s^−1^ and *v*_t_ = 2.90 km s^−1^ and *v*_m_ = 3.18 km s^−1^. Stronger interatomic bonds and less lattice anharmonicity are reflected in the higher Debye temperature of 348 K that results from the higher sound velocities. Furthermore greater thermal robustness and resistance to structural weakening at high temperatures are confirmed by the enhanced melting temperature of 877 K. Overall the thermodynamic indicators listed in Table 3 demonstrate that both A_2_ZrH_6_ (A = Na, K) perovskite hydrides possess sufficient lattice stability to support reversible hydrogen absorption and release. The maximum *θ*_D_ and *T*_m_ values of K_2_ZrH_6_ suggest improved resistance to thermal degradation and more stable H_2_ storage kinetics while Na_2_ZrH_6_ offers a balance between lattice flexibility and thermal integrity encouraging for efficient H_2_ diffusion.

**Table 3:** Thermal parameters calculated for A_2_ZrH_6_ (A = Na, K) perovskite hydrides

Compounds	*v*_l_ (km s^−1^)	*v*_t_ (km s^−1^)	*v*_m_ (km s^−1^)	*θ*_D_ (K)	*T*_m_ (K)
Na_2_ZrH_6_	3.04	2.32	2.48	293	668
K_2_ZrH_6_	4.43	2.90	3.18	348	877

## Conclusion

This work presents a comprehensive first principles investigation of A_2_ZrH_6_ (A = Na, K) double perovskite hydrides employing DFT calculations within the CASTEP framework to evaluate their potential for H_2_ storage and clean energy applications. The structural stability of both compounds is confirmed by their cubic *Fm*3̄*m* symmetry crystallization and their thermodynamic stability is further supported by negative formation enthalpies. By proving that both compounds retain structural stability at 300 K AIMD provides more evidence for thermal stability. According to mechanical analysis both compounds have a mechanical character that is primarily brittle and satisfy the Born stability requirements. The optical response shows high static dielectric constants and significant absorption in the ultraviolet area while the electronic study shows semiconducting band gaps of 1.25 and 1.87 eV for Na_2_ZrH_6_ and K_2_ZrH_6_ respectively. The calculated gravimetric H_2_ storage capacities of 4.22 wt% for Na_2_ZrH_6_ and 3.45 wt% for K_2_ZrH_6_ approach the U.S. DOE targets while their corresponding desorption temperatures of 441.39 K and 258.91 K respectively underscore their promising potential for next-generation solid state H_2_ storage technologies. These results suggest that A_2_ZrH_6_ hydrides offer a promising combination of high H_2_ storage capacity, thermodynamic viability and structural integrity making them great candidates for future H_2_ storage and energy delivery systems.

## Conflicts of interest

All authors declare that they have no conflicts of interest.

## Data Availability

Data relevant to this study is available upon request.
